# Pulmonary Symptoms as the First Manifestation of Crohn’s Disease

**DOI:** 10.7759/cureus.9379

**Published:** 2020-07-25

**Authors:** Gagandeep Kaur, Oluwaseyi Olayinka, Mary S Chacho, Hani El-Fanek, Ramapriya Vidhun

**Affiliations:** 1 Pathology, Danbury Hospital, Danbury, USA

**Keywords:** pulmonary manifestations, crohn’s disease, garnuloma

## Abstract

Crohn’s disease is a granulomatous systemic illness with extra-intestinal manifestations. Of these extra-intestinal manifestations, lung involvement (0.4%) is the rare manifestation. Bronchopulmonary signs and symptoms are underrecognized, so suspicion should be high when granulomas are seen in lung biopsies. We report the case of a 27-year-old female who presented with bilateral pleuritic chest pain and shortness of breath. Chest X-ray showed left lung masses measuring up to 3.3 cm in the greatest dimension with right mid lung nodular opacity. Given the possibility of metastatic disease, positron emission tomography CT (PET-CT) scan was done, which showed activity in multiple liver lesions and multiple bilateral lung nodules. Both liver and lung biopsies were done, which showed multiple necrotizing and non-necrotizing granulomas. The patient was discharged home on antibiotics and antifungals. Few months later, she presented with loose stools and abdominal pain. CT scan of the abdomen and pelvis showed diffuse colonic wall thickening concerning for colitis. Colonoscopy showed ulcerated mucosa involving multiple parts of the colon. Biopsy of the colon showed mild to moderate acute colitis with submucosal non-necrotizing epithelioid granulomas, consistent with Crohn’s disease.

## Introduction

Crohn’s disease (CD) is a granulomatous systemic illness with intestinal and extra-intestinal manifestations. Arthritis, erythema nodosum, pyoderma gangrenosum, and hepatobiliary disease, such as liver granulomas and primary sclerosing cholangitis, are among the most common extra-intestinal manifestations. Although subclinical alterations in lung function have been demonstrated in at least half of adults with CD, clinically significant lung disease is extremely rare with an overall prevalence of 0.4% [1]. CD can involve the tracheobronchial tree, lung parenchyma, and pleura causing latent asymptomatic to severe clinical manifestations. Among all the pulmonary manifestations, the presence of granulomas should be most concerning for CD. Other differential diagnosis of granulomas in the lungs, including histoplasmosis, atypical mycobacterium, sarcoidosis, and connective tissue disorder (hypersensitivity pneumonitis), should be ruled out. CD and sarcoidosis have many similar clinical manifestations; however, their co-existence is rarely seen [2]. The etiology of CD is not well understood, and vascular localization of granulomatous inflammation suggests that the intestinal microvasculature contains an early element in the pathogenesis of CD [3]. We report a case of CD presenting for the first time with pulmonary symptoms.

## Case presentation

**Figure 1:**
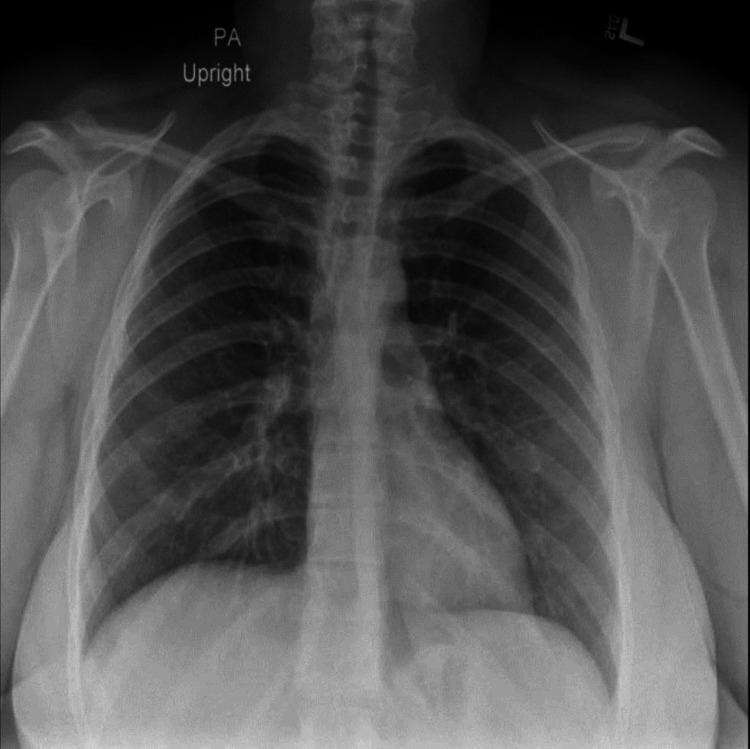
Chest X-ray showing bilateral pulmonary nodules

We present the case of a 27-year-old female patient with no significant past medical history. She presented with bilateral pleuritic chest pain and progressive shortness of breath. History was significant for bird exposure as she was working in the poultry industry. An elevated D-dimer was found on initial laboratory evaluation. CT angiogram of the chest with contrast showed no definitive pulmonary embolus. Chest X-ray and CT scan were done that showed left lung masses up to 3.3 cm in greatest dimension, and right mid lung nodular opacity was noted (Figure 1). 

**Figure 2:**
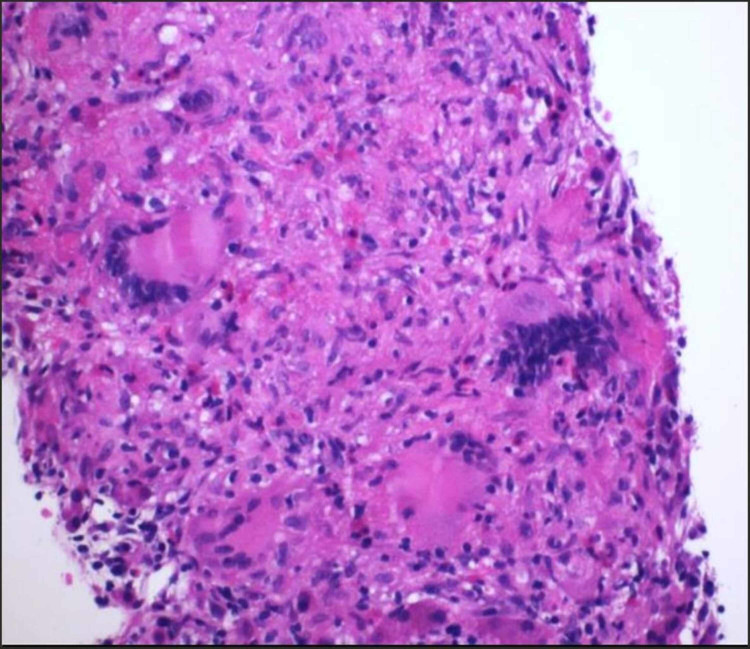
Hematoxylin and eosin stained (×200) liver biopsy showing non-necrotizing granulomas

Positron emission tomography CT (PET-CT) skull base to mid-thigh was performed to look for metastatic disease. PET-CT scan showed activity in multiple liver lesions and multiple bilateral lung nodules. Given the possibility of lung metastasis, a liver biopsy was done that showed benign liver parenchyma with multiple necrotizing and non-necrotizing granulomas (Figure 2). 

**Figure 3:**
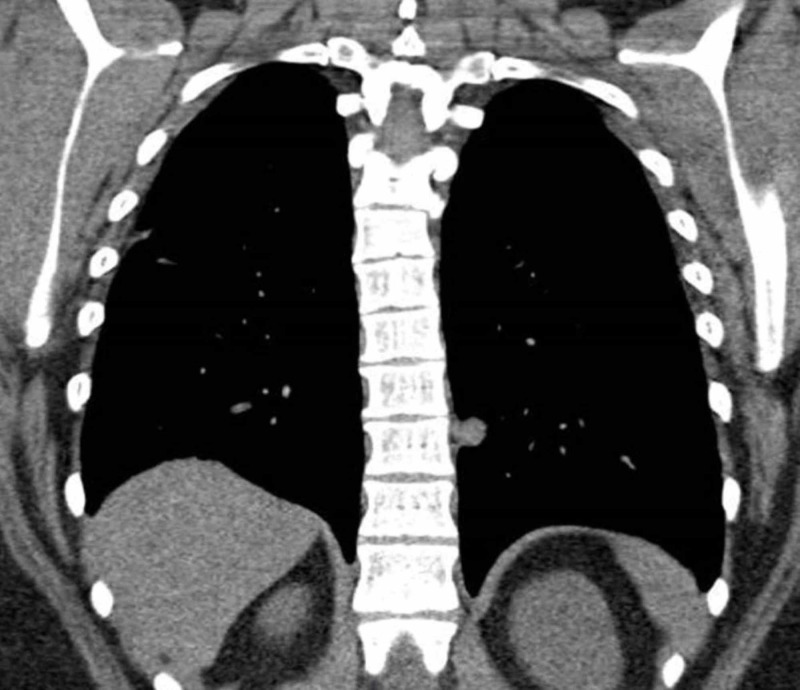
Positron emission tomography CT (PET-CT) scan showing bilateral pulmonary nodules

Stains for microorganisms including acid-fast bacilli (AFB) and Gomori methenamine silver (GMS, fungal organisms) were negative. The patient was discharged home on 10 days of antibiotics with follow-up CT scan of the chest to assess pulmonary nodules. After three months, follow-up CT scan of the chest without contrast was done that showed interval increase in size and number of bilateral pulmonary nodules (Figure 3). 

**Figure 4:**
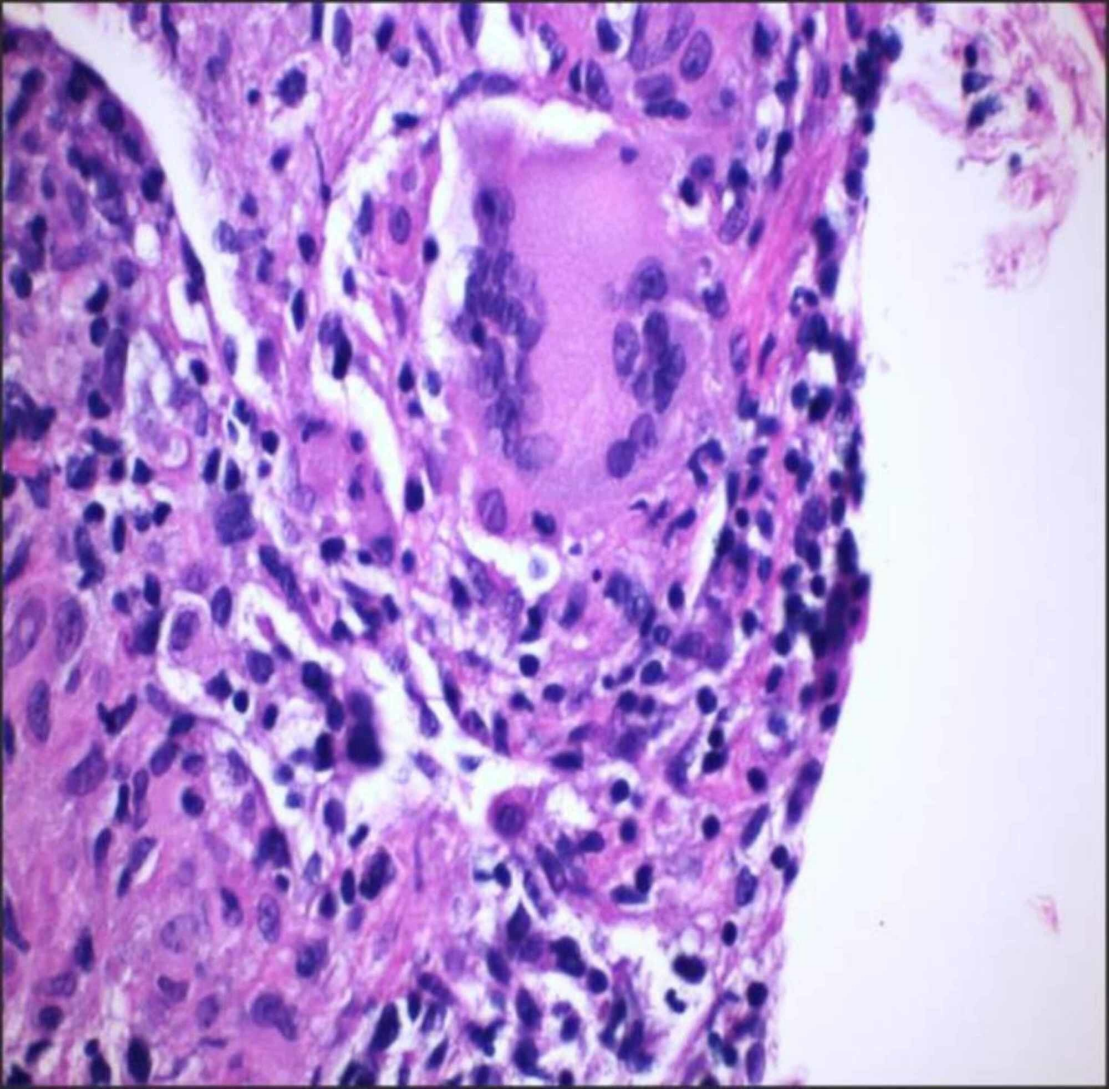
Hematoxylin and eosin stained (×200) lung biopsy showing non-necrotizing granulomas

Transbronchial biopsy of the left lung lower lobe was performed, which showed fragments of benign lung tissue with mild cellular interstitial inflammation, non-necrotizing granulomata, and organizing pneumonia (Figure 4). AFB and GMS stains were negative for organisms. The patient was started on antifungals given the history of exposure to the birds in addition to elevated eosinophils and IgE levels. The patient subsequently improved. However, she presented few months later with two-week history of loose stools and abdominal pain. Bilateral tender erythematous nodules were also noted in the lower extremities. She had no recent change in diet or travel. Gastrointestinal polymerase chain reaction (PCR) panel was negative. 

**Figure 5:**
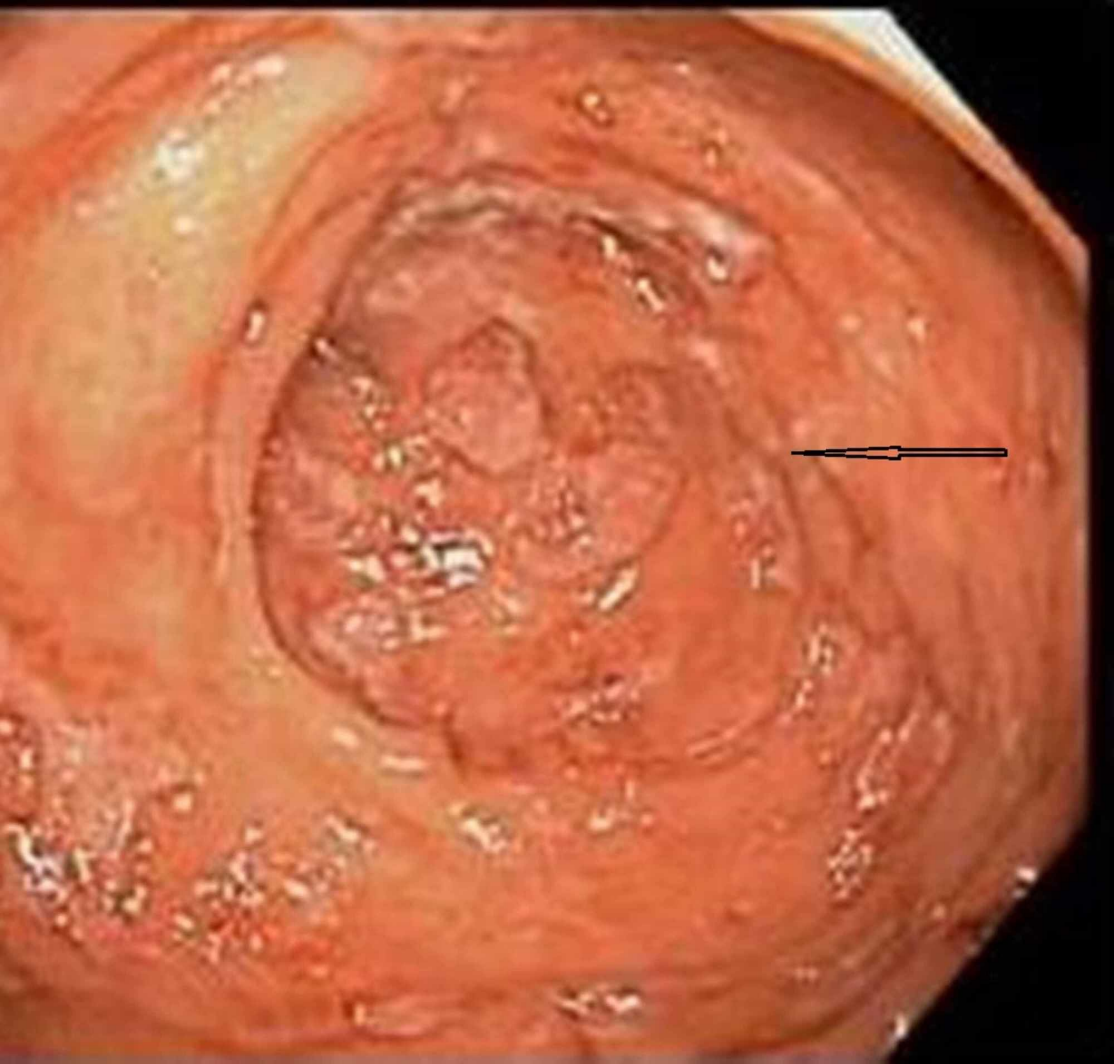
Colonoscopy image showing ulceration

CT scan of the abdomen and pelvis revealed diffuse colonic wall thickening concerning for colitis and focal area of wall thickening with apple core lesion at the junction of the descending and sigmoid colon concerning for colonic malignancy. Colonoscopy was done that showed ulcerated mucosa involving terminal ileum, cecum, ascending colon, hepatic flexure, transverse colon, splenic flexure, descending colon, and sigmoid colon (Figure 5). 

**Figure 6:**
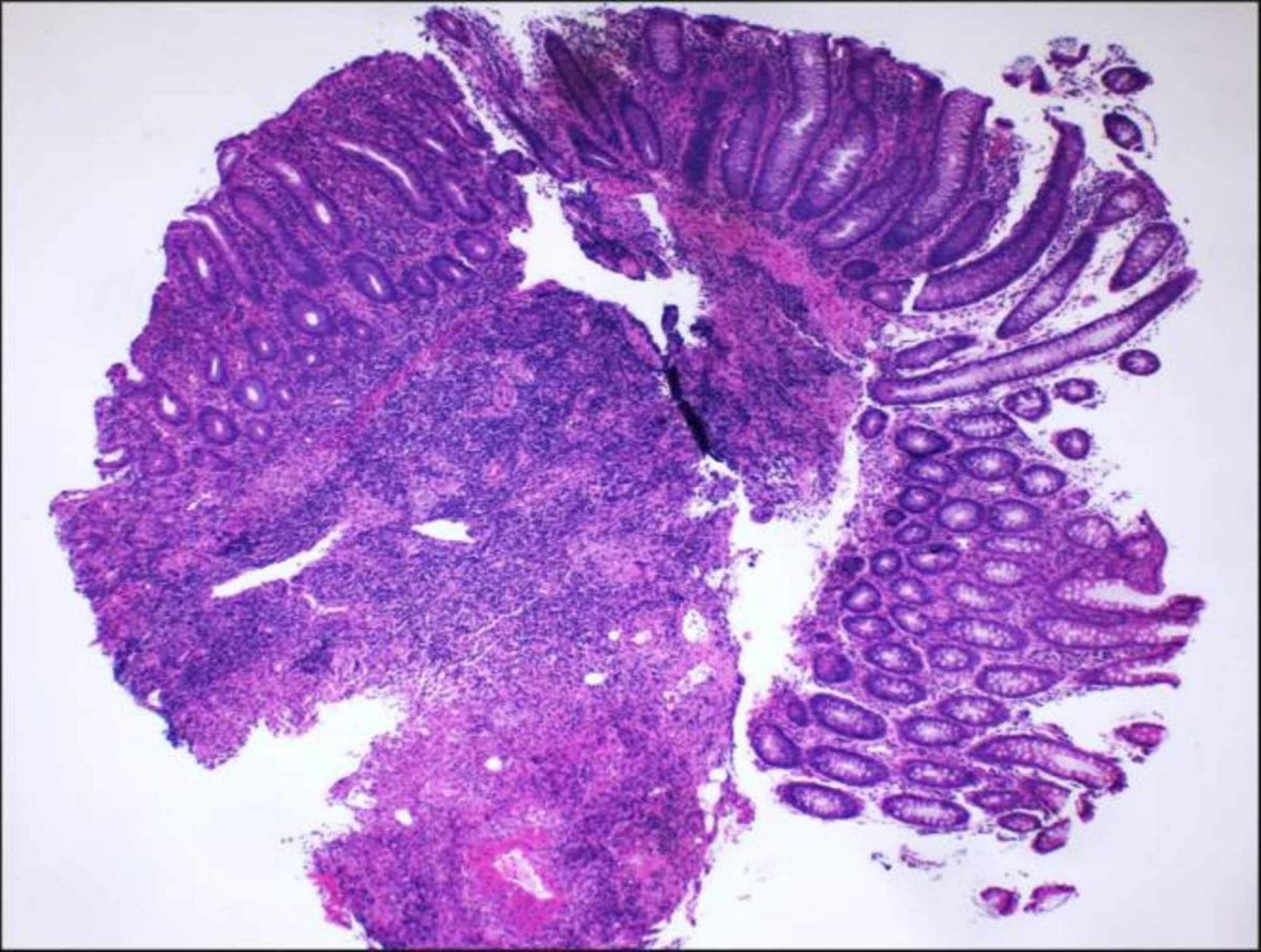
Hematoxylin and eosin stained (×40) section from the colon showing ulceration

**Figure 7:**
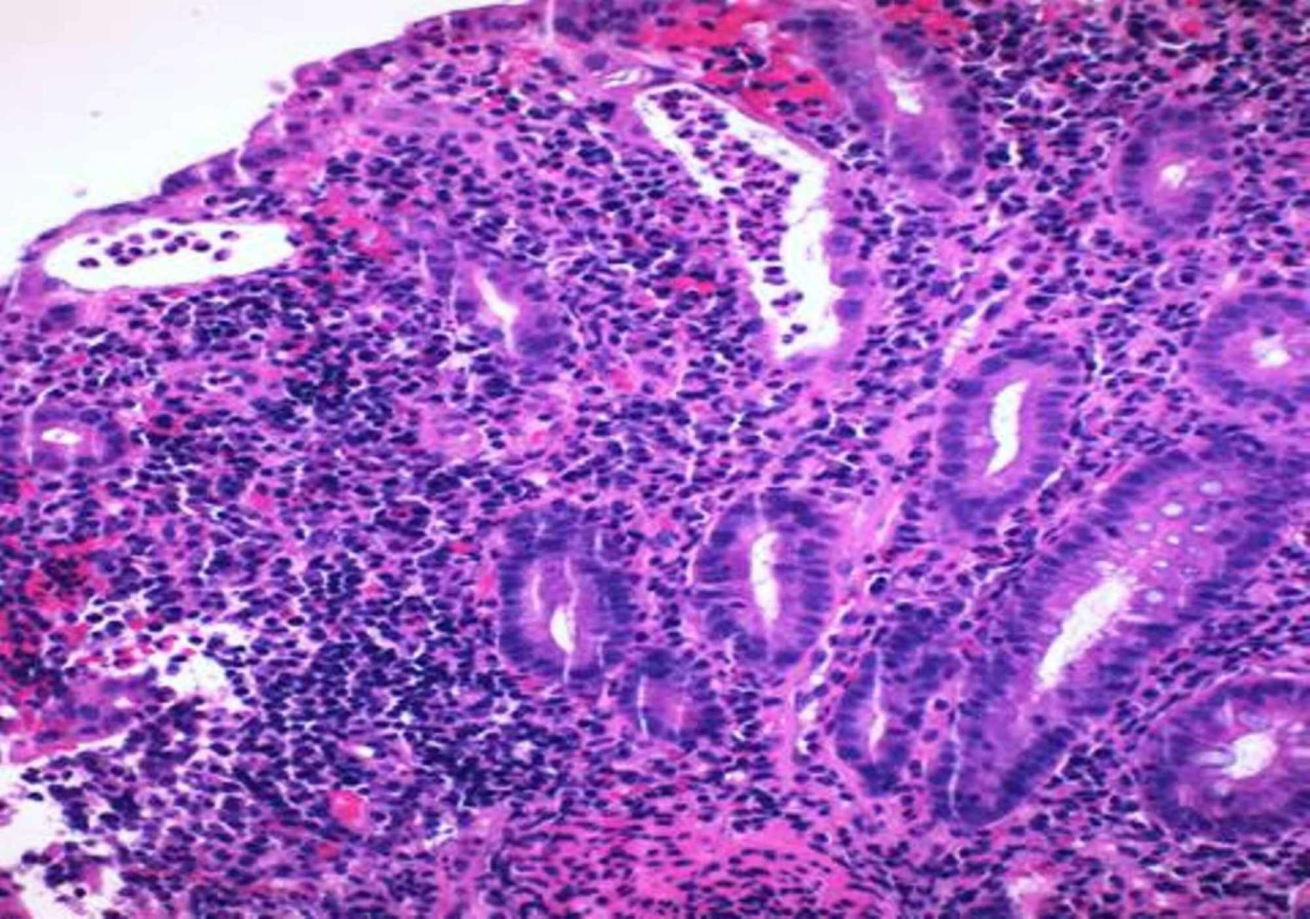
Hematoxylin and eosin stained (×200) section from the colon showing cryptitis and crypt abscess

**Figure 8:**
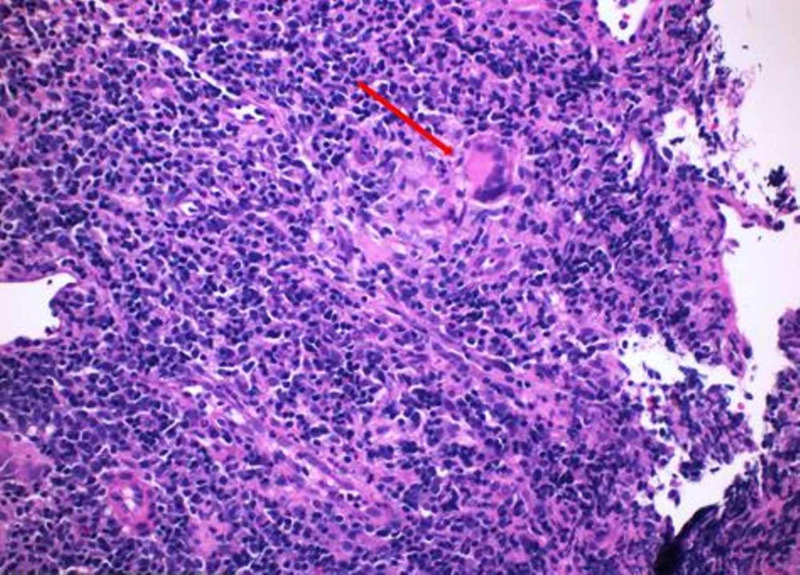
Hematoxylin and eosin stained section (×200) from the colon showing non-necrotizing granuloma

**Figure 9:**
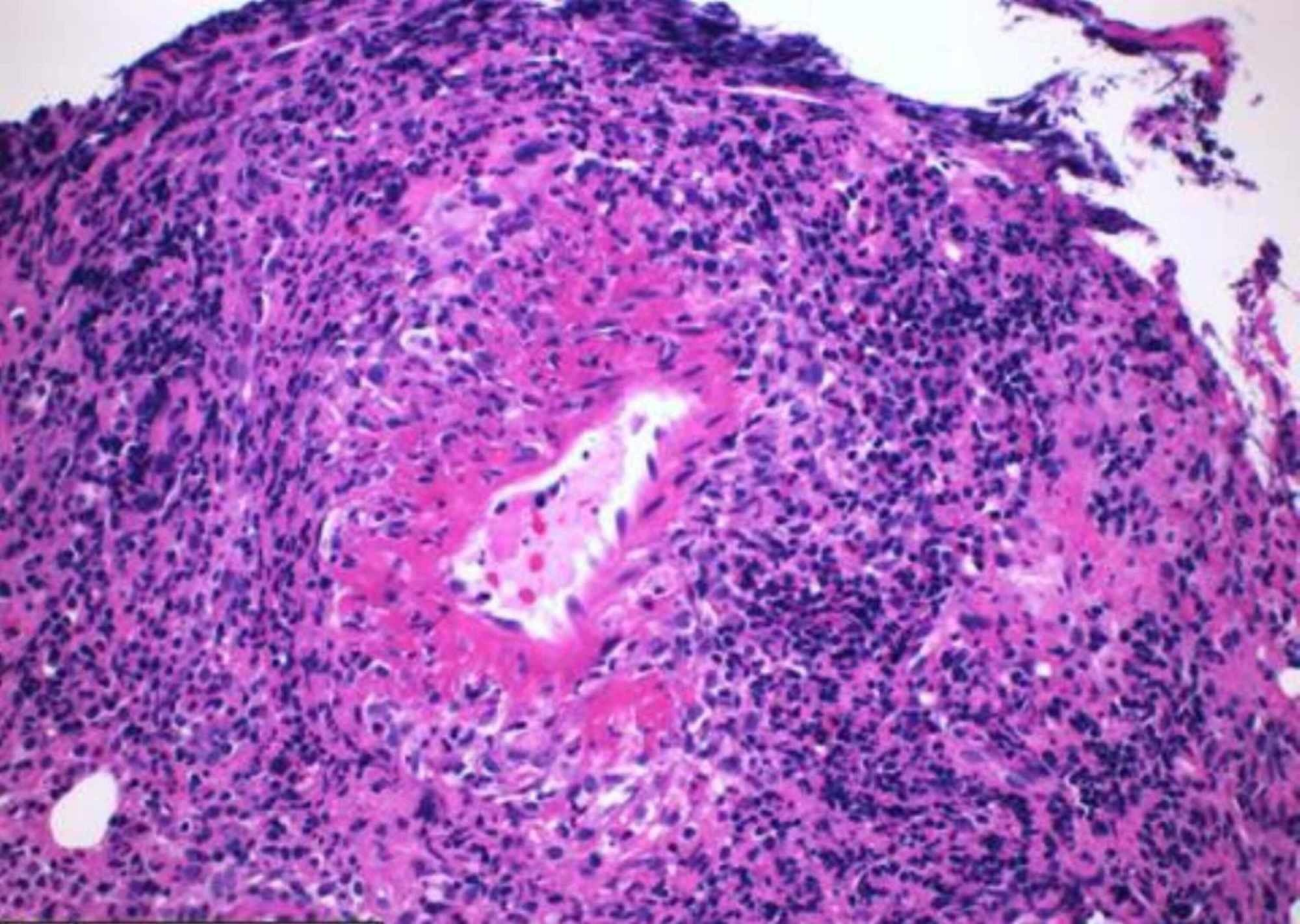
Hematoxylin and eosin stained section (×200) from the colon showing vasculitis

Biopsies were taken that revealed mild to moderate acute colitis with submucosal non-necrotizing granulomas, consistent with CD (Figures 6-8). Granulomatous vasculitis of the blood vessels was also noted (Figure 9). Stains for microorganisms including GMS (fungal) and AFB performed were negative. The patient was started on steroids, Humira (adalimumab) and 5 mercaptopurine, and is doing well currently. 

## Discussion

CD is a granulomatous systemic disorder with intestinal and extra-intestinal manifestations. Extra-intestinal manifestations, which can involve nearly any organ in the body, have been reported in 25%-40% of patients with inflammatory bowel disease [1]. Of all the extra-intestinal manifestations, lung involvement is rare. A number of mechanisms have been proposed by which lung can be involved in patients with CD such as same embryological origin of lung and gastrointestinal tract, similar immune systems, and adverse pulmonary effects of CD drugs.

Lung manifestations vary and patients with lung involvement often have to undergo complex investigations. The possible lung manifestations include interstitial lung disease, including bronchiolitis obliterans with organizing pneumonia (BOOP), unspecified interstitial lung disease, pulmonary infiltrates and eosinophilia, noncaseating granulomatous inflammation, and fibrosis. Airways can also be involved, such as tracheal obstruction (subglottic stenosis), chronic bronchitis, bronchiectasis, chronic bronchial suppuration, and chronic bronchiolitis [1,4,5]. These complications are underrecognized. Identifying these manifestations will help in early diagnosis of CD and will facilitate appropriate and timely management of these patients.

In most cases of CD-related pulmonary manifestations, the development of lung disease either parallels that of intestinal disease activity or occurs after the onset of intestinal disease. In our case, the patient’s pulmonary disease preceded the onset of gastrointestinal symptoms. This is a rare presentation of CD and very few cases of such presentation have been reported in the literature [4,5]. This is in concordance with a case series by Camus et al., which showed in few cases pulmonary manifestations predated the bowel disease by several months and up to several years [5].

## Conclusions

Lung involvement (0.4%) is the rare manifestation of CD. Clinical suspicion should be high when granulomas are seen in lung biopsies, and this will facilitate appropriate and timely management of patient with CD.
